# Resveratrol Inhibits Inflammatory Responses via the Mammalian Target of Rapamycin Signaling Pathway in Cultured LPS-Stimulated Microglial Cells

**DOI:** 10.1371/journal.pone.0032195

**Published:** 2012-02-21

**Authors:** Lian-Mei Zhong, Yi Zong, Lin Sun, Jia-Zhi Guo, Wei Zhang, Ying He, Rui Song, Wen-Min Wang, Chun-Jie Xiao, Di Lu

**Affiliations:** 1 School of Life Science, Yunnan University, Kunming, Yunnan, China; 2 Department of Neurology, the First Affiliated Hospital of Kunming Medical University, Kunming, Yunnan, China; 3 Department of Anatomy, Kunming Medical University, Kunming, Yunnan, China; 4 Department of Cardiology, the Second Affiliated Hospital of Kunming Medical University, Kunming, Yunnan, China; Universidad Federal de Santa Catarina, Brazil

## Abstract

**Background:**

Resveratrol have been known to possess many pharmacological properties including antioxidant, cardioprotective and anticancer effects. Although current studies indicate that resveratrol produces neuroprotection against neurological disorders, the precise mechanisms for its beneficial effects are still not fully understood. We investigate the effect of anti-inflammatory and mechamisms of resveratrol by using lipopolysaccharide (LPS)-stimulated murine microglial BV-2 cells.

**Methodology/Principal Findings:**

BV-2 cells were treated with resveratrol (25, 50, and 100 µM) and/or LPS (1 µg/ml). Nitric oxide (NO) and prostaglandin E2 (PGE2) were measured by Griess reagent and ELISA. The mRNA and protein levels of proinflammatory proteins and cytokines were analysed by RT-PCR and double immunofluorescence labeling, respectively. Phosphorylation levels of PTEN (phosphatase and tensin homolog deleted on chromosome 10), Akt, mammalian target of rapamycin (mTOR), mitogen-activated protein kinases (MAPKs) cascades, inhibitor κB-α (IκB-α) and cyclic AMP-responsive element-binding protein (CREB) were measured by western blot. Resveratrol significantly attenuated the LPS-induced expression of NO, PGE2, inducible nitric oxide synthase (iNOS), cyclooxygenase-2 (COX-2), tumor necrosis factor-α (TNF-α), interleukin-1β (IL-1β) and nuclear factor-κB (NF-κB) in BV-2 cells. Resveratrol increased PTEN, Akt and mTOR phosphorylation in a dose-dependent manner or a time-dependent manner. Rapamycin (10 nM), a specific mTOR inhibitor, blocked the effects of resveratrol on LPS-induced microglial activation. In addition, mTOR inhibition partially abolished the inhibitory effect of resveratrol on the phosphorylation of IκB-α, CREB, extracellular signal-regulated kinase 1/2 (ERK1/2), c-Jun N-terminal protein kinase (JNK), and p38 mitogen-activated protein kinase (p38 MAPK).

**Conclusion and Implications:**

This study indicates that resveratrol inhibited LPS-induced proinflammatory enzymes and proinflammatory cytokines via down-regulation phosphorylation of NF-κB, CREB and MAPKs family in a mTOR-dependent manner. These findings reveal, in part, the molecular basis underlying the anti-inflammatory properties of resveratrol.

## Introduction

Microglia, the resident immune cells in the brain, serves the first line of defense when injury or disease occurs and play a homeostatic role in the central nervous system (CNS) [1]. Although activated microglia scavenge dead cells from the CNS and secrete different neurotrophic factors for neuronal survival [2], [3], it is believed that severe activation causes various autoimmune responses leading to neuronal death and brain injury [4], [5]. Activation of microglia and consequent release of proinflammatory and/or cytotoxic factors such as tumor necrosis factor-α (TNF-α), interleukin-1β (IL-1β), nitric oxide (NO), prostaglandin E2 (PGE2), reactive oxygen species (ROS), inducible nitric oxide synthase (iNOS), and cyclooxygenase-2 (COX-2) are believed to contribute to neuronal damage, particularly in neurodegenerative diseases [6], [7], [8], [9]. Subsequently, the damaged neurons release toxic soluble factors, which in turn induce microglial activation termed as reactive microgliosis [10].

It has recently been suggested that the activation of microglia can increase neurotoxicity through the production of proinflammatory and cytotoxic factors in neuron-glia cultures treated with lipopolysaccharide (LPS), β-amyloid, glutamate, and arachidonate [11]. One of these widely used stimuli is LPS, a bacterial endotoxin used to study experimentally induced infection, inflammation, or tissue damage, as well as the biochemistry of inflammatory responses. LPS activates nuclear factor-κB (NF-κB), cyclic AMP responsive element-binding protein (CREB) and mitogen-activated protein kinases (MAPKs) family, which are classified into at least three components: extracellular signal-regulated kinases 1/2 (ERK 1/2), c-Jun N-terminal kinase (JNK), and p38 MAPK [12] and which have been implicated in the release of immune-related cytotoxic factors such as iNOS, COX-2, and proinflammatory cytokines [7], [12].

The mammalian target of rapamycin (mTOR) is a serin/threonin protein kinase with a central role in the regulation of cell growth and proliferation, as well as of physiological processes such as transcription, mRNA turnover and translation, ribosomal biogenesis, vesicular trafficking, autophagy and cytoskeletal organization [13]. mTOR exists in two functionally distinct complexes called mTORC1 and mTORC2. mTORC1, composed of mTOR, mLST8/GβL (G protein β-subunit like protein) and raptor (regulatory associated protein of mTOR) is sensitive to rapamycin, unlike mTORC2 which is composed of mTOR, mLST8/GβL and rictor [14]. In freshly isolated human monocytes and primary myeloid dendritic cells (DCs), mTOR activation inhibits the production of proinflammatory cytokines while it enhances the release of the anti-inflammatory cytokine by blocking NF-κB activation and increasing STAT3 activity [15]. Thus, the mTOR pathway might have opposite roles in the immune system [16]. Recent studies indicated that mTOR selectively controls microglial activation in response to proinflammatory cytokines and appears to play a crucial role in microglial viability [17], mediates iNOS induction during hypoxia in the BV-2 microglial cell line [18], and reduces microglia/macrophages activation and increases the number of surviving neurons after brain injury [19], suggesting a key role in the control of microglial functions.

**Figure 1:**
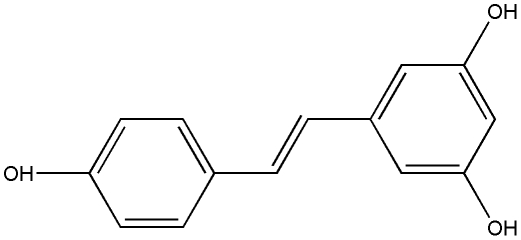
Chemical structure of resveratrol.

Resveratrol (3, 4, 5-trihydroxy-trans-stilbene) is a natural non-flavonoid polyphenolic found in grapes, red wine, mulberries, knotweed, peanuts and other plants (Figure 1) [20]. Although these plants and their extracts have been used for various therapeutic purposes by ancient cultures, resveratrol itself was first described in 1940 as a phenolic component of the medicinal herb hellebore [21]. There are numerous reports in the literature show that resveratrol dampens inflammation in arthritis and immune responsiveness in autoimmune diseases [22], [23], suppresses angiogenesis and metastasis in several cancers [24], and inhibits ROS products and platelet aggregation in cardiovascular diseases [25], [26]. Resveratrol can penetrate the blood-brain barrier to exert strong neuroprotective effects in vivo [21]. In addition to these beneficial actions, resveratrol has been noted for its anti-inflammatory activities. It can attenuate the activation of immune cells and the subsequent synthesis and release of proinflammatory mediators through the inhibition of the transcriptional factors such as NF-κB and activator protein-1 (AP-1) [27]. Although current studies indicate that resveratrol produces neuroprotection against neurological disorders, such as Alzheimer disease, Parkinson disease, and Huntington disease [28], the precise mechanisms for its beneficial effects are still not fully understood.

This study examined the effects of resveratrol on LPS-stimulated inflammatory responses in microglia and the potential role of mTOR in this process. To this end, a fuller understanding of the molecular mechanism of microglial activation is clearly desirable in delineating the therapeutic target molecules to reduce the brain inflammation and resulting neuronal injury or death in neurodegenerative diseases.

## Results

### Resveratrol and/or rapamycin do not affect the viability of BV-2 cells

**Figure 2:**
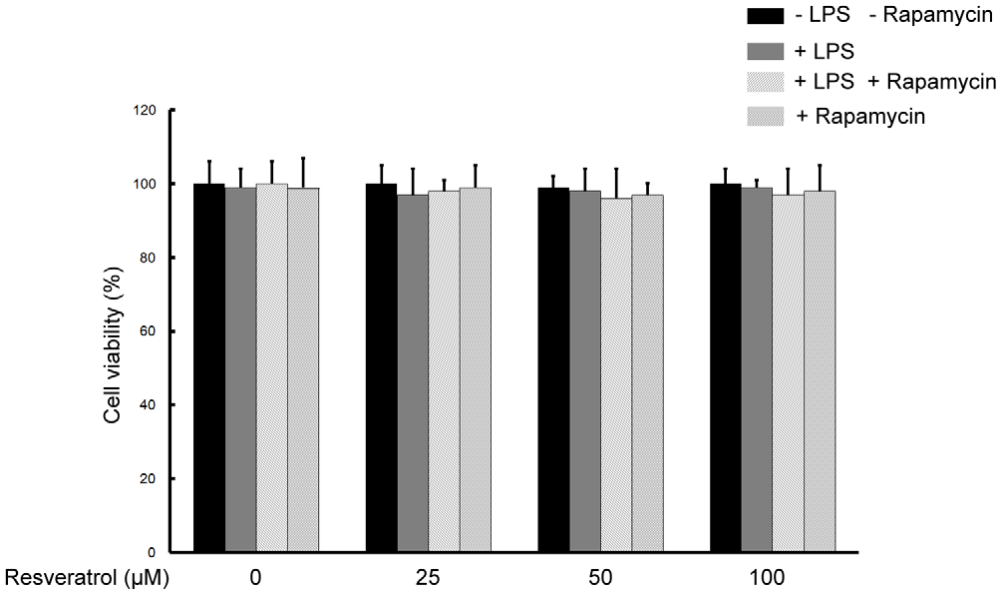
Effects of resveratrol on the cell viability of BV-2 microglial cells. BV-2 cells were treated with 25, 50, and 100 µM of resveratrol without LPS treatments or with 1 µg/ml LPS or with 10 nM rapamycin treatments for 18 h. BV-2 cells viabilities were measured and expressed as mean ± SEM for three independent experiments.

The cytotoxicity of resveratrol and rapamycin was evaluated in the presence or absence of LPS by MTT assay. Resveratrol and/or rapamycin did not decrease the viability of the BV-2 microglial cells when they were incubated with or without LPS (1 µg/ml) in the presence or absence of resveratrol (25, 50, and 100 µM) and/or rapamycin (10 nM) for 18 h. Hence, resveratrol and/or rapamycin exerted no significant cytotoxicity on BV-2 microglial cells (Figure 2).

### Resveratrol activates the mTOR signaling pathway in BV-2 cells

**Figure 3:**
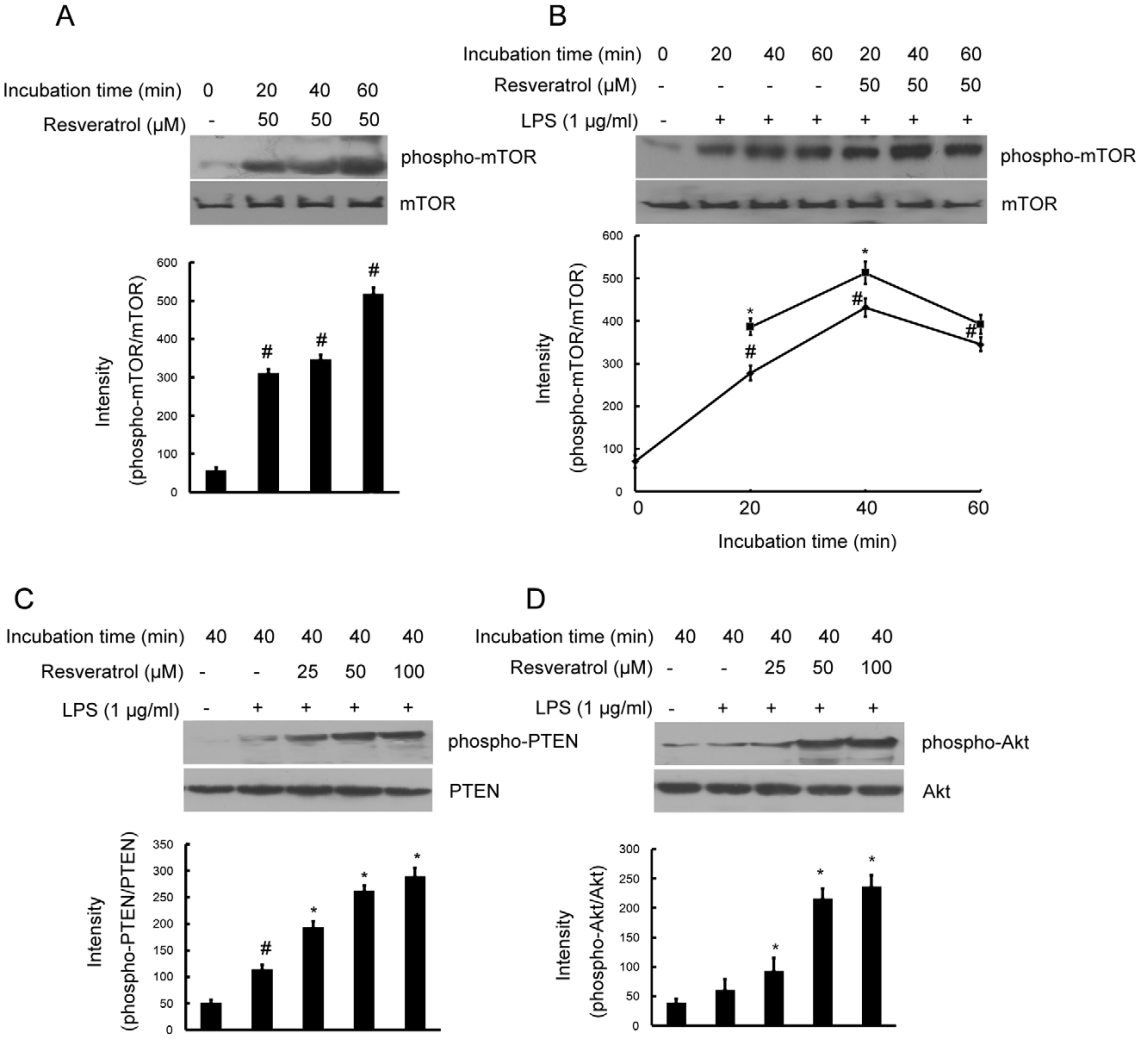
Resveratrol activates the Akt/mTOR signaling pathway in BV-2 microglial cells. Panel A shows that BV-2 cells were treated with resveratrol (50 µM) for the indicated times, Panel B shows that BV-2 cells were pre-treated with resveratrol (50 µM) for 1 h, then exposed to LPS (1 µg/ml) for the indicated times and Panels C and D shows that BV-2 cells were pre-treated with resveratrol (25, 50, and 100 µM) for 1 h, then exposed to LPS (1 µg/ml) for 40 min. Various treated BV-2 cell lysates (50 µg protein) were prepared and subjected to Western blot analysis by using antibodies specific for PTEN and phospho-PTEN, Akt and phospho-Akt, mTOR and phospho-mTOR as described in the methods. The relative protein levels were quantified by scanning densitometry and normalized to total Akt. The values shown are mean ± SEM of data from three independent experiments. ^#^Significant compared with control alone, *p*<0.05. *Significant compared with LPS alone, *p*<0.05. ^▵^Significant compared with resveratrol+LPS, *p*<0.05.

To identify the signaling pathways that are activated by resveratrol in cultured microglial BV-2 cells. We investigated whether resveratrol could activate the mTOR signaling pathway and affect LPS-induced mTOR phosphorylation. Incubation of BV-2 cells with resveratrol (50 µM) significantly induced phosphorylation of serine residue 2881 of mTOR in time-dependent manner (Figure 3A). Furthermore, when the cells were treated with resveratrol (50 µM) for 1 h before LPS (1 µg/ml) stimulation, mTOR phosphorylation was elevated initially and further increased steadily during the 1 h incubation with LPS. The levels of phospho-mTOR were always significantly higher in the cells pretreated with resveratrol compared with the cells treated with LPS only (Figure 3B).

PTEN (phosphatase tensin homolog on chromosome 10) and Akt is an effective modulator of mTOR, so we further examined whether resveratrol affects PTEN and Akt phosphorylation induced by LPS. When the cells were treated with resveratrol (25, 50, and 100 µM) for 1 h before LPS (1 µg/ml) stimulation for 40 min, resveratrol significantly increased phosphorylation of PTEN, so it downregulated PTEN activity by its phosphorylation (Figure 3C), and the level of phospho-Akt was always significantly higher in dose-dependent manner in the cells pretreated with resveratrol compared with the cells treated with LPS only (Figure 3D).

### mTOR is involved in resveratrol-inhibited releases of NO and PGE2 induced by LPS in BV-2 cells

**Figure 4:**
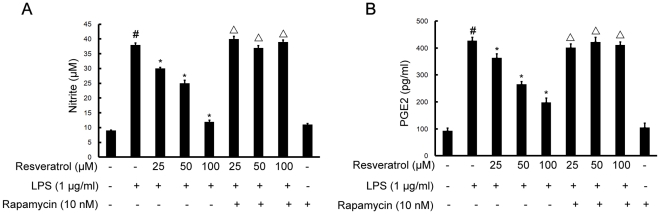
mTOR is required for resveratrol-inhibited expression of NO (A) and PGE2 (B) induced by LPS in BV-2 cells. Approximately 1×10^6^ cells/ml were seeded in six-well plates and incubated until 80% confluency. Cells were pre-treated with resveratrol (25, 50, and 100 µM) for 1 h in the absence or presence of rapamycin (10 nM), then exposed to LPS (1 µg/ml) for 8 h. The values shown are mean ± SEM of data from three independent experiments. ^#^Significant compared with control alone, *p*<0.05. ^*^Significant compared with LPS alone, *p*<0.05. ^▵^Significant compared with resveratrol+LPS, *p*<0.05.

Treatment of BV-2 cells with LPS (1 µg/ml) caused a significantly increase in NO and PGE2 releases in comparison with untreated controls by Griess reagent and enzyme-linked immunosorbent assay (ELISA), after 8 h exposure to LPS. Resveratrol (25, 50, and 100 µM) inhibited the LPS-induced production of NO and PGE2 over the concentration range used here. However, following the pretreatment of cells with 10 nM rapamycin (1 h before), a specific mTOR inhibitor, significantly reversed resveratrol-downregulated levels of NO and PGE2 induced by LPS (Figures 4A and B).

### mTOR is required for resveratrol-inhibited expression of iNOS and COX-2 proteins and mRNA induced by LPS in BV-2 cells

**Figure 5:**
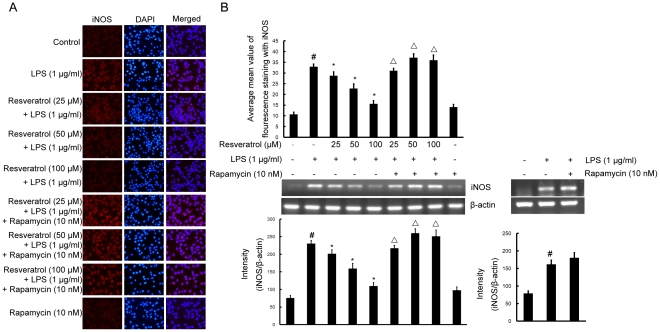
mTOR is required for resveratrol-inhibited expression of iNOS protein and mRNA induced by LPS in BV-2 cells. Panel A shows the immunofluorenscence images for protein expression of iNOS and Panel B shows the corresponding mRNA data. The relative mRNA level was quantified by scanning densitometry and normalized to β-actin mRNA. Note the up-regulated protein and mRNA expression of iNOS by LPS is suppressed by different concentrations of resveratrol; however, in cells pretreated with mTOR inhibitor rapamycin, the suppressive effect of resveratrol is abrogated. The values shown are mean ± SEM of data from three independent experiments. ^#^Significant compared with control alone, *p*<0.05. ^*^Significant compared with LPS alone, *p*<0.05. ^▵^Significant compared with resveratrol+LPS, *p*<0.05.

**Figure 6:**
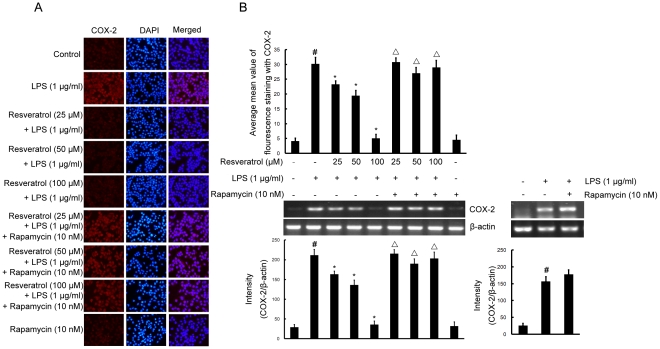
mTOR is required for resveratrol-inhibited expression of COX-2 protein and mRNA induced by LPS in BV-2 cells. Panel A shows the immunofluorenscence images for protein expression of COX-2 and Panel B shows the corresponding mRNA data. The relative mRNA level was quantified by scanning densitometry and normalized to β-actin mRNA. Note the up-regulated protein and mRNA expression of COX-2 by LPS is suppressed by different concentrations of resveratrol; however, in cells pretreated with mTOR inhibitor rapamycin, the suppressive effect of resveratrol is abrogated. The values shown are mean ± SEM of data from three independent experiments. ^#^Significant compared with control alone, *p*<0.05. ^*^Significant compared with LPS alone, *p*<0.05. ^▵^Significant compared with resveratrol+LPS, *p*<0.05.

To investigate the effect of resveratrol on iNOS and COX-2, the expression of both proinflammatory enzymes was examined by double immunofluorescence labeling and RT-PCR assay. BV-2 cells were stimulated with LPS (1 µg/ml) for 4 h which resulted in increase of the protein and mRNA levels of iNOS and COX-2. Pre-treatment with resveratrol (25, 50, and 100 µM) inhibited iNOS and COX-2 protein and mRNA levels significantly (Figures 5 and 6), compared with LPS-treated cells. However, following the pretreatment of cells with 10 nM rapamycin (1 h before), resveratrol could not inhibit upregulation of the protein and mRNA levels of iNOS and COX-2 induced by LPS (Figures 5 and 6).

### mTOR is involved in resveratrol-attenuated expression of the proinflammatory cytokines TNF-α and IL-1β in LPS stimulated BV-2 cells

**Figure 7:**
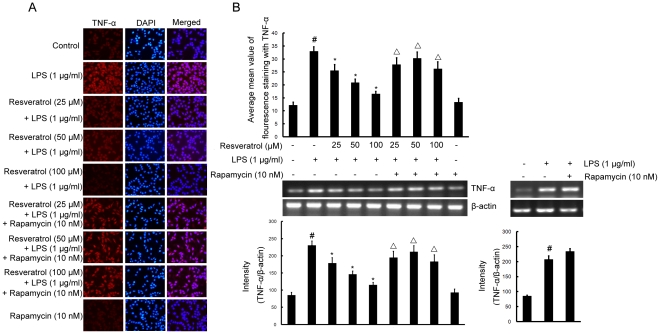
mTOR is involved in resveratrol-attenuated the production of the proinflammatory cytokine TNF-α at the transcriptional and translational levels in BV-2 cells. Panel A shows the immunofluorenscence images for protein expression of TNF-α and Panel B shows the corresponding mRNA data. The relative mRNA level was quantified by scanning densitometry and normalized to β-actin mRNA. Note the up-regulated protein and mRNA expression of TNF-α by LPS is suppressed by different concentrations of resveratrol; however, in cells pretreated with mTOR inhibitor rapamycin, the suppressive effect of resveratrol is abrogated. The values shown are mean ± SEM of data from three independent experiments. ^#^Significant compared with control alone, *p*<0.05. ^*^Significant compared with LPS alone, *p*<0.05. ^▵^Significant compared with resveratrol+LPS, *p*<0.05.

**Figure 8:**
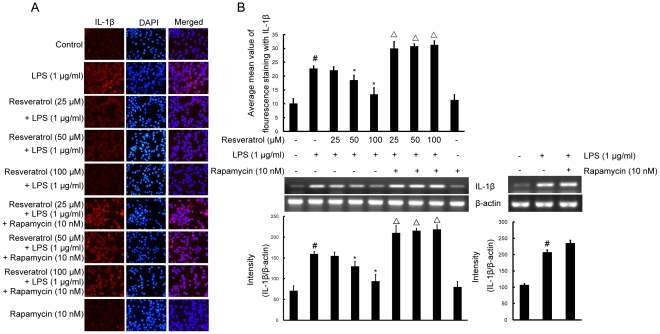
mTOR is involved in resveratrol-attenuated the production of the proinflammatory cytokine IL-1β at the transcriptional and translational levels in BV-2 cells. Panel A shows the immunofluorenscence images for protein expression of IL-1β and Panel B shows the corresponding mRNA data. The relative mRNA level was quantified by scanning densitometry and normalized to β-actin mRNA. Note the up-regulated protein and mRNA expression of IL-1β by LPS is suppressed by different concentrations of resveratrol; however, in cells pretreated with mTOR inhibitor rapamycin, the suppressive effect of resveratrol is abrogated. The values shown are mean ± SEM of data from three independent experiments. ^#^Significant compared with control alone, *p*<0.05. ^*^Significant compared with LPS alone, *p*<0.05. ^▵^Significant compared with resveratrol+LPS, *p*<0.05.

To investigate whether resveratrol represses the production of TNF-α and IL-1β and whether *mTOR* is involved in this process in BV-2 cells, cells were stimulated with LPS (1 µg/ml) for 4 h in the presence or absence of resveratrol (25, 50, and 100 µM). After treatment with LPS, the protein levels of the cytokines in BV-2 cells were evaluated by immunofluorescence labeling which showed that TNF-α as well as IL-1β immunoexpression was noticeably enhanced by LPS stimulation. Pretreatment with resveratrol resulted in a drastic decrease in cytokine expression. However, in BV-2 cells subjected to pretreatment with 10 nM rapamycin (1 h before), TNF-α and IL-1β immunofluorescence intensity was obviously increased (Figures 7A and 8A).

To further investigate whether the inhibitory effect of resveratrol on TNF-α and IL-1β production is due to the reduced expression of cognate genes, the effect of resveratrol on mRNA expression of TNF-α and IL-1β was assessed in LPS-stimulated BV-2 cells. As shown in Figures 7B and 8B, the mRNA expression of these inflammatory mediators was very low or hardly detectable in unstimulated BV-2 cells. However, BV-2 cells expressed high levels of TNF-α and IL-1β mRNA when stimulated with LPS (1 µg/ml; 4 h). More importantly, resveratrol suppressed LPS-induced expression of these genes. In parallel to the double immunofluorescence labeling, pretreatment of BV-2 cells with rapamycin significantly reversed resveratrol-downregulated levels of TNF-α and IL-1β mRNA (Figures 7B and 8B).

### mTOR pathway is linked to resveratrol-suppressed LPS-induced expression of NF-κB/RelA protein, phosphorylation of IκB-α and CREB, and protects against LPS-induced activation in BV-2 cells

**Figure 9:**
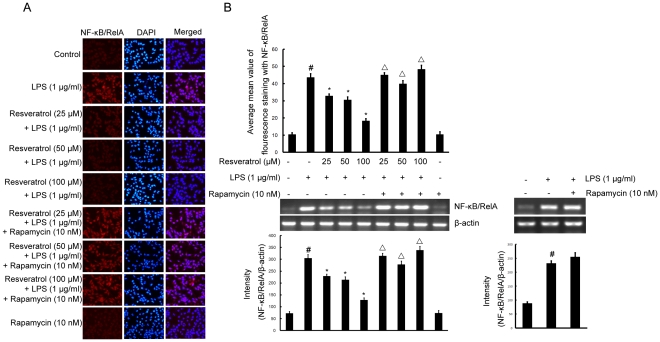
mTOR is required for resveratrol-inhibited expression of NF-κB/RelA protein and mRNA induced by LPS in BV-2 cells. Panel A shows the immunofluorenscence images for protein expression of NF-κB/RelA and Panel B shows the corresponding mRNA data. The relative mRNA level was quantified by scanning densitometry and normalized to β-actin mRNA. Note the up-regulated protein and mRNA expression of NF-κB/RelA by LPS is suppressed by different concentrations of resveratrol; however, in cells pretreated with mTOR inhibitor rapamycin, the suppressive effect of resveratrol is abrogated. The values shown are mean ± SEM of data from three independent experiments. ^#^Significant compared with control alone, *p*<0.05. ^*^Significant compared with LPS alone, *p*<0.05. ^▵^Significant compared with resveratrol+LPS, *p*<0.05.

**Figure 10:**
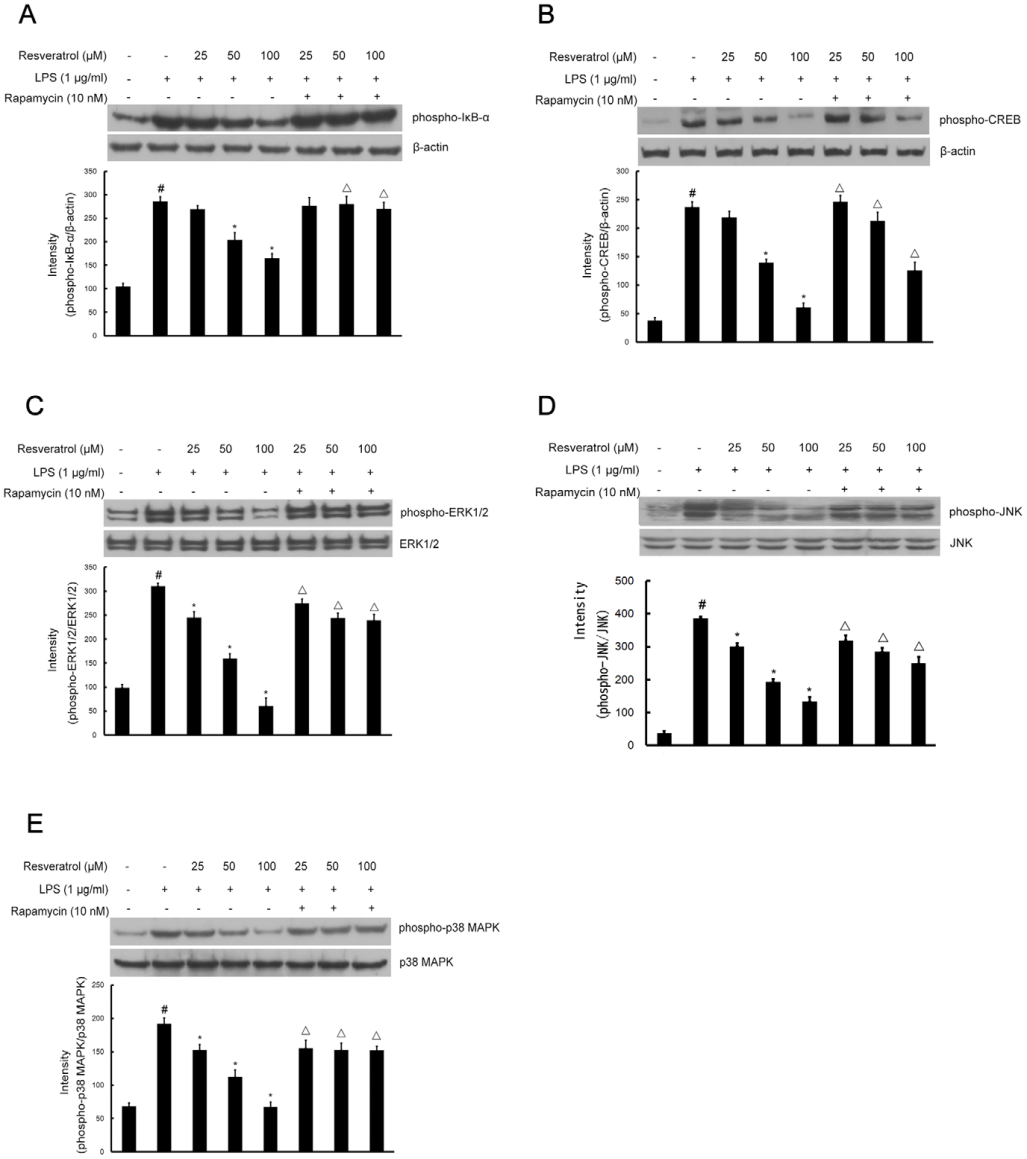
Inhibition of phosphorylation of IκB-α, CREB, and MAPKs signaling by resveratrol is mTOR-dependent during BV-2 cells activation by LPS. Approximately 1×10^6^ cells/ml were seeded in six-well plates and incubated until 80% confluency. Cells were pre-treated with resveratrol (25, 50, and 100 µM) for 1 h in the absence or presence of rapamycin (10 nM), then exposed to LPS (1 µg/ml) for 30 min. Cell lysates (50 µg protein) were prepared and subjected to Western blot analysis by using antibodies specific for phosphorylated forms of IκB-α, CREB, ERK1/2, JNK and p38 MAPK (shown as phospho-IκB-α, etc.) as described in the methods. Equivalent loading of cell lysates was determined by reprobing the blots with anti-β-actin, total ERK1/2, JNK or p38 MAPK antibodies. The relative protein levels were quantified by scanning densitometry and normalized to β-actin, total ERK1/2, JNK or p38 MAPK. The values shown are mean ± SEM of data from three independent experiments. ^#^Significant compared with control alone, *p*<0.05. ^*^Significant compared with LPS alone, *p*<0.05. ^▵^Significant compared with resveratrol+LPS, *p*<0.05.

To further elucidate the mechanisms of resveratrol on the inhibition of expression of iNOS, COX-2, and proinflammatory cytokines in microglia, we next examined the effect of resveratrol on NF-κB and CREB, the two major transcription factors involved in the expression of these inflammatory mediators. NF-κB activation includes IκB-α's degradation through phosphorylation and a subsequent nuclear NF-κB translocation. We determined whether the resveratrol inhibitory effect was through the blockade of NF-κB activation in BV-2 cells. As shown in Figure 9, pre-treatment of resveratrol (25, 50, and 100 µM) significantly suppressed the LPS (1 µg/ml; 4 h) induced expression levels of NF-κB/RelA mRNA and protein, as well as phosphorylation of IκB-α in a mTOR-dependent manner (Figure 10A).

CREB is the physiological substrate for MAPKs and stress-activated protein kinases-1 (MSK1), which is activated by ERK and p38 MAPK-mediated signaling in response to LPS. We next determined whether resveratrol regulates the phosphorylation of CREB and whether such an effect, if any, is mediated through the mTOR signaling pathway in BV-2 cells. As demonstrated in Figure 10B, CREB activation was markedly stimulated by LPS; additionally the activation was significantly inhibited by treatment with resveratrol at 50 and 100 µM; nonetheless, pre-treatment with rapamycin at 10 nM had increased the phosphorylation of CREB most drastically.

### Inhibition of MAPKs signaling by resveratrol is mTOR-dependent during BV-2 cells activation by LPS

The effect of resveratrol on MAPKs, which are upstream signaling molecules in inflammatory reactions, was examined in LPS-stimulated BV-2 cells. Western blot analysis was carried out using the phospho- or total forms of antibodies against the three MAPKs, including ERK1/2, JNK, and p38 MAPK. It was observed that resveratrol at different concentrations 25, 50, and 100 µM significantly decreased the LPS-stimulated phosphorylation of ERK1/2 at 30 min, respectively, whereas it had no effect on the expression level of ERK1/2 in LPS-stimulated BV-2 cells (Figure 10C). Likewise, resveratrol at all concentrations significantly suppressed the phosphorylation of JNK and p38 MAPK, respectively, but did not affect the expression levels of JNK and p38 MAPK in LPS-stimulated BV-2 cells (Figures 10D and E). To determine the upstream regulators of MAPKs signaling in resveratrol-inhibited activation of BV-2 cells by LPS, BV-2 cells were pretreated with 10 nM rapamycin (1 h before) and, very interestingly, this reversed the effect of resveratrol-inhibited phosphorylation of ERK1/2, JNK and p38 MAPK (Figures 10C, D and E).

## Discussion

Over-activation of microglia contributes to neurodegenerative processes through the production of various neurotoxic factors including free radicals and proinflammatory cytokines [29]. Therefore, the inhibition of microglial activation could reduce neuronal cell death. In fact, a number of anti-inflammatory agents, which inhibit microglial activation or production of proinflammatory mediators under the central nervous system disease conditions, attenuate neuronal degeneration [30]. The present study was undertaken to examine the pharmacological and biological effects of resveratrol on the production of inflammatory mediators in murine BV-2 microglia stimulated with LPS. To further understand the molecular mechanism of resveratrol activity in microglia, we investigated the effects of resveratrol on the mRNA and protein expression levels of iNOS, COX-2 and cytokines (TNF-α, IL-1β), the activation of transcription factor NF-κB and CREB, the activation of the MAPKs family, and if mTOR signaling pathway might be involved in resveratrol's action on activated BV-2 cells. The results of this study indicated that resveratrol activated the mTOR signaling pathway and induced mTOR phosphorylation in a time-dependent manner in BV-2 cells. Similar to resveratrol, LPS also induced slightly mTOR phosphorylation in BV-2 cells. However, the levels of phospho-mTOR significantly increased 40 min after the addition of LPS. Thus, it appears that the delay in mTOR activation initially allows LPS to activate MAPKs and induce an acute inflammatory response. In contrast, when the cells were treated with resveratrol before LPS stimulation, mTOR phosphorylation was elevated initially and further increased during LPS exposure. Hence, the proinflammatory response to LPS was suppressed by resveratrol from the beginning, resulting in inhibition of LPS-induced MAPKs activity and activation of transcription factor NF-κB and CREB. PTEN is originally identified as tumor suppressor gene mutated in a large percentage of human cancers [31]. It is considered to be a key negative regulator of the Akt/mTOR signaling, which is well known as a prosurvival pathway, suggesting perspective molecular target for anti-cancer research [32]. Moreover, several reports have suggested anti-inflammatory activity of PTEN [33], [34]. Here we further identified that resveratrol inactivates PTEN protein by its phosphorylation in LPS-stimulated BV-2 cells. Moreover, the presence of resveratrol significantly increased phosphorylation of Akt in LPS-stimulated BV-2 cells. Thus, we suggest that the modulatory effect of resveratrol on PTEN/Akt/mTOR signaling is critical for its inhibition of proinflammatory mediators and cytokines expression in LPS-activated BV-2 cells.

COX-1 is constitutively expressed in most tissues, while COX-2 is induced by an array of stimuli including cytokines, LPS, and growth factor in microglia and astrocytes [7]. COX-2 is the key enzyme in the formation of PGE2 which appear to be an important source of PGE2 during inflammatory conditions [35]. Microglial cells in the healthy brain do not express iNOS, but they become activated to produce iNOS and to release a large amount of NO following ischemic, traumatic, neurotoxic or inflammatory damage [36]. Therefore, any substance that can attenuate expression of iNOS and COX-2 could be beneficial for delaying the progression of neurological disorders. In the present study, resveratrol (25, 50, and 100 µM) significantly inhibited the production of NO, PGE2, iNOS and COX-2 in a dose-dependent manner in LPS-stimulated BV-2 microglial cells, suggesting possible beneficial effects of resveratrol by attenuation of activation of microglial cells and subsequent inflammatory neurotoxins. More importantly, we have shown that pretreatment of BV-2 cells with rapamycin (10 nM), a mTOR inhibitor, effectively reversed the protective effects of resveratrol as evidenced by the reduced expression levels of NO, PGE2, iNOS and COX-2 that were comparable to that induced by LPS alone. These results suggest that mTOR plays an important role in the inhibition of NO, PGE2, iNOS and COX-2 following resveratrol-protection against LPS-induced microglial activation.

TNF-α and IL-1β are two main proinflammatory cytokines that are produced by activated microglia during CNS inflammation. In the CNS, a number of stimuli, such as LPS, amyloid-β and traumatic brain injury have been shown to abundantly produce TNF-α and IL-1β [37], [38]. Overproduction of proinflammatory cytokines from activated microglial cells has a detrimental effect on neuronal cells. This study investigated whether resveratrol inhibits LPS-induced production of proinflammatory cytokines in BV-2 cells. Resveratrol inhibited LPS-induced production of TNF-α and IL-1β in a dose-dependent manner. Meanwhile, Pre-treatment with resveratrol significantly inhibited TNF-α mRNA levels, and resveratrol notablely inhibited dose-dependently IL-1β mRNA levels, compared with LPS-treated control. Furthermore, we have shown that pretreatment of cells with rapamycin followed by LPS+resveratrol, TNF-α and IL-1β protein and mRNA expression levels were markedly increased compared with that of the cells treated with LPS+resveratrol. The findings support that mTOR participates in resveratrol-inhibited expression of proinflammatory cytokines.

The transcriptional regulation of NO, PGE2, iNOS, COX-2 and inflammatory cytokines, such as TNF-α and IL-1β, is a tightly controlled event. A variety of transcription factors, including NF-κB and CREB, is known to be involved in the transcriptional regulation of these inflammatory mediators [39]. In un-stimulated cells, NF-κB is retained in the cytoplasm by binding to IκB-α. The processes of NF-κB activation include degradation through phosphorylation and a subsequent nuclear translocation of the RelA subunit of NF-κB. The molecular mechanisms underlying anti-inflammatory effect of resveratrol, which showed the most potent anti-inflammatory activity, were further studied. The present results have shown that NF-κB/RelA level, markedly increased by LPS stimulation in BV-2 cells, was effectively reversed by resveratrol treatment in a mTOR-dependent manner. Concomitantly, pre-incubation of LPS stimulated cells with rapamycin reversed the resveratrol-induced phosphorylation of IκB and CREB. These results indicated that the inhibition of resveratrol on the expression of NO, PGE2, iNOS, COX-2 and proinflammatory cytokines is partially through the suppression of NF-κB/RelA expression, the phosphorylation of IκB-α and CREB in a mTOR-dependent manner in LPS-stimulated BV-2 cells.

MAPKs family has been shown to play important roles in LPS-induced iNOS, COX-2, and proinflamatory cytokines expression in many types of cells [40], [41], [42]. It also has been reported that LPS-induced proinflammatory cytokines expression is mediated by MAPKs signal transduction pathway in BV-2 cells [43]. Therefore, we investigate the effect of resveratrol on activation (phosphorylation) of three MAPKs induced by LPS in BV-2 cells. The results of this study indicate that resveratrol inhibits LPS-increased activation of MAPKs, including ERK1/2, JNK, and p38 MAPK, within 30 min after stimulation, whereas resveratrol decreased LPS-induced activation of MAPKs, which was accompanied by alterations in NO, PGE2, iNOS, COX-2, and proinflammatory cytokines. This result is inconsistent with a recent study [27]. The discrepancy may be due to differences in cell origin and experimental conditions (Our data indicate that resveratrol-inhibited activation of MAPKs occurs at higher concentrations). Additionally, rapamycin partly reversed the effect of resveratrol-inhibited phosphorylation of ERK1/2, JNK and p38 MAPK.

In conclusion, this investigation demonstrates that resveratrol significantly attenuates overactivation of microglial cells by repressing expression levels of neurotoxic proinflammatory mediators and cytokines via activation of mTOR signaling pathway. These results suggest the potential of resveratrol as an anti-inflammatory drug candidate.

## Methods

### Cells and treatments

The mouse microglial cell line BV-2 was developed in the laboratory of Dr Blasi at the University of Perugia and was a generous gift of Dr Cheng-gang Zou (School of Life Science, Yunnan University, Kunming, China). Cells were cultured in Dulbecco's modified Eagle's medium (DMEM; Gibco/BRL, Gaithersburg, MD, USA) containing 2% fetal bovine serum (Hyclone, Logan, UT, USA) and antibiotics (100 IU/ml penicillin and 100 µg/ml streptomycin; Sigma, St. Louis, MO, USA) at a density not exceeding 5×10^5^ cells/ml and maintained at 37°C in a humidified incubator with 5% CO_2_. To harvest BV-2 cells, cells were trypsinized (0.25% trypsin/EDTA in phosphate-buffered saline (PBS); Sigma, St. Louis, MO, USA), then centrifuged (400 g for 10 min) and resuspended in serum-free DMEM. Cells were counted with a hemocytometer and trypan blue staining (0.4% trypan blue in PBS; Sigma) showed more than 98% of the cells retained viability. Cells (approximately 1×10^6^ cells/ml) were seeded in six-well plates before being subjected to treatments. Resveratrol (Purity>99%; Kunming Pharmaceutical Corporation, Kunming, China) at 25, 50, and 100 µM was added 1 h before LPS (1 µg/ml) (from *Escherichia coli*, Sigma) stimulation. This time point was chosen to minimize the possibility of any direct interactions between resveratrol and LPS. Cell incubations ranged from 20 min–18 h, as indicated in the text.

### Cell cytotoxicity test

The cells were seeded in a 24-well dish (1×10^6^ cells/ml) for 24 h before being exposed to resveratrol (25, 50, and 100 µM), resveratrol with LPS (1 µg/ml), resveratrol with rapamycin (10 nM), or resveratrol with LPS+rapamycin for 18 h. MTT solution (0.5 mg/ml) was then added to each well and the cells were incubated for 2 h at 37°C and in 5% CO_2_. Subsequently, the supernatant was removed and the formation of farmazan was solubilized with dimethyl sulfoxide (DMSO) and measured at 540 nm with a microplate reader.

### Assay of NO production

Various treated BV-2 cells were plated in 48-well plates at a density of 1×10^6^ cells/ml for 8 h. NO production was monitored by measuring the nitrite content in culture medium as previously described [7]. The isolated supernatant was mixed with an equal volume of Griess reagent (0.1%N-1-naphthylethylenediamine dihydrochloride and 1% sulphanilamide in 5% phosphoric acid) and incubated at room temperature for 10 min. Absorbance was measured at 550 nm in a microplate reader. Sodium nitrite was used as a standard.

### Determination of PGE2 production

Various treated BV-2 cells were treated for 8 h to permit cytokine production. The PGE2 concentration in the culture medium was quantified using a competitive enzyme immunoassay kit (R&D Systems Inc., MN, USA) in accordance with the manufacturer's instructions. The production of PGE2 was measured relative to that observed after control treatment.

### Double-immunofluorescence labeling assay

BV-2 cells derived from various treatments were fixed with 4% paraformaldehyde in 0.1 M phosphate buffer (PB) for 15 min. After rinsing with PBS, the coverslips with adherent cells were used for double immunofluorescence labeling. BV-2 cells were incubated with DAPI (dilution 1∶50,000; Sigma) plus goat anti-mouse iNOS (dilution 1∶500; Santa Cruz Biotechnology, Santa Cruz, CA, USA), goat anti-mouse COX-2 (dilution 1∶500; Santa Cruz Biotechnology), goat anti-rabbit TNF-α polyclonal antibody (dilution 1∶500; Chemicon, Temecula, CA,USA), goat anti-rabbit IL-1β (dilution 1∶500; Chemicon), or goat anti-rabbit NF-κB/RelA (dilution 1∶500; Santa Cruz Biotechnology). Subsequently, the cells were incubated with TRITC-conjugated secondary antibody (Santa Cruz Biotechnology) for 1 h at room temperature. For negative controls, a set of culture slides was incubated under similar conditions without the primary antibodies. All images were captured with a fluorescence microscope (80i; Nikon, Tokyo, Japan). The results are representative of three independent experiments.

### Reverse transcription-polymerase chain reaction (RT-PCR) analysis

After incubation, media was removed and BV-2 cells were washed with phosphate-buffered saline (PBS) twice. Total RNA was prepared from BV-2 cells by using the Trizol® reagent (Invitrogen Corporation, Carlsbad, CA, USA) according to the manufacturer's protocol. cDNA was prepared using reverse transcriptase originating from the Superscript™-III kit (Invitrogen) with 2.5 µg total RNA and oligo dT. The sequences of PCR primers were as follows: iNOS, sense: 5′- CTGCAGCA CTTGGATCAGGAACCTG -3′, antisense: 5′- GGGAGTAGCCTGTGTGCACCTGGAA -3′; COX-2, sense: 5′-TTGAAGACCAGGAGTACAGC-3′, antisense: 5′-GGTACAGTTCCATGACA TCG-3′; TNF-α, sense: 5′-CGTCAGCCGATTTGCTATCT-3′, antisense: 5′-CGGACTCCGCAAA GTCTAAG-3′; IL-1β, sense: 5′-GCCCATCCTCTGTGACTCAT-3′, antisense: 5′-AGGCCACAGG TATTTTGTCG-3′; NF-κB/RelA, sense: 5′-GCGTACACATTCTGGGGAGT-3′, antisense: 5′-CCGAAGCAGGAGCTATCAAC-3′; β-actin, sense: 5′-AGCCATGTACGTAGCCATCC-3′, antisense: 5′-GCTGTGGTGGTGAAGCTGTA-3′. PCR amplification of the resulting cDNA template was conducted by using the following conditions for 45 (TNF-α, IL-1β, NF-κB/RelA and β-actin), 36 (COX-2) or 27 (iNOS) cycles. After an initial denaturation step at 95°C for 15 min, temperature cycling was initiated. Each cycle consisted of denaturation at 94°C for 15 sec, annealing at 60°C for 25 sec, and elongation at 72°C for 20 sec (TNF-α, IL-1β, NF-κB/RelA and β-actin). For COX-2, after an initial denaturation step at 95°C for 5 min, temperature cycling was initiated. Each cycle consisted of denaturation at 94°C for 30 sec, annealing at 57°C for 45 sec, and elongation at 72°C for 30 sec. For iNOS, after an initial denaturation step at 95°C for 5 min, temperature cycling was initiated. Each cycle consisted of denaturation at 94°C for 45 sec, annealing at 60°C for 45 sec, and elongation at 70°C for 1 min. PCR products were analyzed on 1% agarose gels and stained with 1 mg/ml ethidium bromide. Images were captured with a Gel Doc 2000 image analyzer (Bio-Rad, Richmond, CA, USA). The results are representative of three independent experiments.

### Western blot analysis

BV-2 cells were incubated in a medium without 2% FBS for at least 4 h before treatments. They were harvested with ice-cold PBS and centrifuged at 16 000× *g* for 5 min at 4°C. Stimulated cells were lysed in ice-cold lysis buffer [62.5 mM Tris–HCl, pH 6.8, 25% glycerol, 2% sodium dodecyl sulphate (SDS), 0.01% bromphenol blue and 5% β-mercaptoethanol]. Cell lysates were centrifuged at 16 000×*g* for 5 min at 4°C, then the supernatants were collected. Protein content was determined by using the BCA protein assay (Pierce, Rockford, IL, USA). Equal amounts of total cellular protein (50 µg) were loaded per lane onto 10% SDS-polyacrylamide gel electrophoresis (SDS-PAGE) and transferred onto immunoblot polyvinylidene difluoride membranes (Chemicon). The membranes were blocked with 5% non-fat milk in Tris-buffered saline containing 0.1% Tween 20 (TBS-T) for 2 h and incubated separately with goat anti-rabbit antibodies for PTEN and phospho-PTEN, Akt and phospho-Akt, mTOR and phospho-mTOR, ERK1/2 and phospho-ERK1/2, JNK and phospho-JNK, p38 MAPK and phospho-p38 MAPK, phospho-IκB-α, phospho-CREB and β-actin antibodies (1∶1000 dilution; Cell Signaling Technology, Danvers, MA, USA) that recognize different molecules under study for overnight at 4°C. The membranes were then washed three times for 15 min with TBS-T, and incubated with a 1∶2000 dilution of horseradish peroxidase-coupled secondary antibodies (Santa Cruz Biotechnology) for 2 h at room temperature. Blots were again washed three times for 5 min each in TBS-T and developed by the ECL® detection system (Santa Cruz Biotechnology). Membranes were exposed to Fuji Medical X-Ray Film (Fuji Photo Film Co., Ltd, Karagawa, Japan).

### Statistical analysis

Statistical analysis of the data was carried out by one way analysis of variance (ANOVA) followed by Scheffe's post hoc test, using SPSS (SPSS Inc., Chicago, IL, USA). Summary data are shown as mean ± SEM (standard error of mean) obtained from three independent experiments. Values of *p*<0.05 were considered significant. (^#^ *p*<0.05, *p<0.05, ^▵^ *p*<0.05).
